# A study of the distribution and abundance of the adult malaria vector in western Kenya highlands

**DOI:** 10.1186/1476-072X-7-50

**Published:** 2008-09-22

**Authors:** Li Li, Ling Bian, Guiyun Yan

**Affiliations:** 1 Department of Political Science and Geography, Old Dominion University, Norfolk, Virginia, USA; 2 Department of Geography, University at Buffalo, Amherst, New York, USA; 3 Program in Public Health, College of Health Sciences, University of California, Irvine, California, USA

## Abstract

**Background:**

A sharp rise in the malaria mortality rate has been observed recently in western Kenya. Malaria is transmitted by mosquito vectors. Malaria control strategies can be more successful if the distribution and abundance of mosquito vectors is predicted. However, how mosquito vectors are distributed in space remain poor understood, and this question is rarely studied using spatial methods. This study aims to provide a better understanding of the distribution and abundance of mosquito vectors. To achieve this objective, spatial and non-spatial methods were employed. The data on the distribution of adult mosquitoes, and mosquito breeding habitats in a study area in western Kenya, and environmental variables were analyzed.

**Results:**

The models developed using spatial methods outperformed the models developed using non-spatial methods. Houses close to locations where mosquito breeding habitats were repeatedly observed had more abundant adult female mosquitoes. Distance to high-order streams was identified as an effective predictor for the distribution of adult mosquitoes.

**Conclusion:**

The spatial method is more effective in modeling the distribution of adult mosquitoes than the non-spatial method. The results of this study can be used to facilitate decision-making related to mosquito surveillance and malaria prevention.

## Introduction

The highland areas in Africa rarely experienced malaria before 1988 [1]. However, a series of explosive seasonal malaria outbreaks has occurred in these areas in the last two decades [2]. These outbreaks caused thousands of deaths of which over 70% were children under the age of five, and the highlands in western Kenya have seen the highest mortality rates [3]. Malaria control is urgently needed for the region.

Malaria is a vector-borne disease, which is transmitted by mosquito vectors. Understanding the spatial distribution of mosquitoes will contribute to the design of malaria-vector control strategies. Many studies have been carried out to improve the understanding of the spatial distribution of mosquito vectors. For example, elevation, temperature, and shape of landscape have been recognized to be related with the development of mosquito vectors [4-6]. The abundance of mosquitoes in human houses has been found to be affected by rainfall [7]. Humidity also has a significant effect on mosquitoes [8,9]. Host availability has long been recognized to have an influence on the distribution of mosquitoes [10]. The survival of mosquito larvae has also been related to the openness and presence of predatory animals in their habitats [11]. It is also believed that certain human activities, such as the deforestation and cultivation of natural swamps, may have created conditions favorable to mosquitoes in highland areas [12].

However, there are three concerns regarding the present mosquito studies. First, the question of whether the spatial stability of mosquito breeding habitats affects the distribution of adult mosquitoes remains unexplored. Second, adult mosquito abundance is traditionally considered to be a function of the availability of human hosts and mosquito breeding habitats [13]. This approach is subject to the omission of some other important factors affecting the distribution of adult mosquitoes, such as moisture level. Third, the non-spatial method is often used to model the distribution of insects. However, data on adult mosquitoes often have a spatial element, which may impose limitations on the non-spatial modeling methods, such as ordinary regression [14].

The objectives of this study are two-fold: (1) to explain the relationship between adult mosquitoes and mosquito breeding habitats, and (2) to model the spatial distribution and abundance of adult mosquitoes. To achieve these objectives, this study employed spatial and non-spatial methods to analyze data on adult mosquitoes, mosquito breeding habitats, and environmental variables collected in a study area in western Kenya.

## Methods

### Study area

The study area, a 4 × 4 km area centered at 0°10' N, 34°45' E, is located in Iguhu Village, Kakamega District of western Kenya, one of the most densely populated districts in Kenya. Frequent malaria outbreaks have been reported in the highlands of the District [15]. The terrain of the study area is typical of the highlands and consists of a mosaic of hills and small basins, with elevations ranging from 1420 to 1540 m. The Yala River runs through the area from east to west. The study area has one long rainy season, one short rainy season and a main dry season. The long rainy season usually occurs from April to June, the short rainy season in October to November, and the main dry season from December to March [16]. The study area contains about 2,500 households and includes a population of over 10,000 people.

### Adult mosquito data

**Figure 1:**
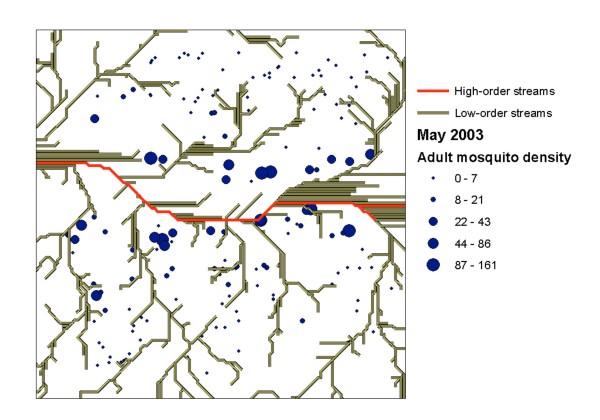
**Locations of the sampled houses, high-order streams and low-order streams. **Each dot represents a sampled house. The size of the dot represents abundance of adult female *An. gambiae *mosquitoes (the number of adult mosquitoes captured in each house).

To determine the distribution and abundance of mosquitoes in human houses, we randomly selected 200 houses in the study area (8% of all the houses in the study area) (Figure 1). A Global Positioning System (GPS) was used to record the coordinates of the selected houses. In May 2003 (long rainy season), the mosquitoes in selected houses were collected using indoor pyrethrum spray collection method, which is a standard adult mosquito survey method. Follow-up laboratory identification was performed on the collected adult mosquitoes to identify their species [17]. Mosquito abundance was measured as the density of female adult *Anopheles gambiae s.l. *at each house (i.e., total number of female adult *An. gambiae s.l. *per house). Only female mosquitoes were included in the study because they are responsible for malaria transmission.

### Mosquito breeding habitat data and data preprocessing

All aquatic habitats in the study area were thoroughly surveyed in February (dry season) and May (long rainy season) from 2003 to 2005. Therefore, the study area was surveyed six times in these three years. The aquatic habitat survey in May 2003 took place a week before the aforementioned adult mosquito survey. Samples from all aquatic habitats (excluding running water and water in containers inside houses) were collected by using a standard dipper (size = 350 ml). Each aquatic habitat was dipped up to 20 times to collect water samples. If a habitat was too small to make 20 dips, it was usually dipped as many times as possible. The presence of *An. gambiae s.l. *larvae in the water samples was then examined. Coordinates taken at the center of all water bodies were recorded using a GPS. Aquatic habitats with the presence of *An. gambiae s.l. *larvae were considered as mosquito breeding habitats.

Using the information collected using the GPS, six point maps showing the location of mosquito breeding habitats were created. Each of these point maps was converted into a raster map with 20 m resolution (this resolution is determined based on the observed surface area of mosquito breeding habitats). On each map, pixels showing the presence and absence of mosquito breeding habitats are coded as 1 and 0, respectively. To examine the spatial stability of habitats, these six maps were then overlaid and added together. This resulted in one raster map, hereafter referred as integrated habitat map. The value of pixels on integrated habitat map represents the number of times that mosquito breeding habitat was observed in locations represented by these pixels during the field surveys. The pixel values of the integrated habitat map range from 0 to 6. If the value of a pixel is larger than 1, this indicates that mosquito breeding habitats are observed repeatedly in the location represented by this pixel.

To obtain information on habitat stability, two maps were used. One is the map showing larval breeding habitats in May 2003, hereafter called May 2003 habitat map, and another is the integrated habitat map. Based on these two maps, six types of habitat locations were identified: (1) locations where habitats were observed in May 2003 (pixel values of May 2003 habitat map = 1), (2) locations where habitats were observed at least once during six surveys (pixel value of the integrated map > 0), (3) locations where habitats were observed only once during six surveys (pixel value of the integrated map = 1), (4) locations where habitats that were observed at least twice during six surveys (pixel value of the integrated map > 1), hereafter called locations with repeatedly observed habitats, (5) locations where habitats were observed at least three times during six surveys (pixel value of the integrated map > 2), and (6) locations where habitats were observed in May 2003 and at least once during other time periods (pixel values of May 2003 habitat map = 1, and pixel value of the integrated map > 2). These six types of locations are not exclusive from each other. Table 1 shows six types of locations and the number of pixels they occupy. Distances from human houses to each of these six types of locations were calculated. Therefore, six distance variables were created.

**Table 1:** Environmental variables that describe hydrological and land surface conditions of the study area.

**Variable**	**Calculation**
Wetness Index	Calculated based on the local upslope contributing area and slope
Distance to High Order Stream	Stream orders are calculated based on the reaches of a stream. A high order stream is reached by streams ordered four or five.
Distance to Low Order Stream	A low order streams is reached by streams ordered one, two, three and four. It also may not be reached by other streams.
Elevation	Derived from Digital Elevation Model
Slope	Calculated as the rate of change in altitude

### Environmental data

Five variables were used to describe the environmental conditions for the adult mosquitoes (Table 1). Among these five variables, wetness index, distance to high-order streams, and distance to low-order streams were used to represent hydrological condition. Hydrological condition of an area is known to have an influence on mosquito breeding habitat, and consequently it may affect the distribution of adult mosquitoes [18].

Wetness index (Ln(A/TanB)), which combines local upslope contributing area and slope, is commonly used to quantify topographic control on hydrological processes, where A is the draining area of the location and B is the slope [19]. For this variable, the larger the value, the higher the soil moisture level. This index is widely used in hydrological studies to represent static equilibrium soil moisture conditions in relation to water flow patterns in an area.

Stream order were used to represent the volume of water in a stream network. Stream order classifies the reaches of a stream according to its relative position in the stream network [20]. The higher the stream order, the greater the volume of water that flows down the stream. At the first order, which is the lowest, streams have the smallest water volume and receive water only from overland flow. These streams normally flow during rainy seasons [20]. When two streams of order j (1, 2...n) join, a stream of order j+1 is formed. High-order streams usually are found at low elevations and have a large water volume. In this study, the highest ordered streams, the fifth- and sixth-order streams, coincide with the Yala River, which is located in the valley of the study area. Streams ordered from five to six were then considered high-order streams, and the others are considered low-order streams. Distance from human houses to high-order streams and distance to low-order streams were used in the study.

In addition to the variables that represent the hydrological condition, slope and elevation were used to represent land surface condition. Although adult mosquitoes tend to fly close to the ground to avoid the wind, they can travel further with the facilitation of wind [21]. The wind may interact with the slope to affect the adult mosquito's dispersal. Therefore, slope angle and elevation were included in the analysis.

These variables were derived from a Digital Elevation Model (DEM) data set. This DEM were calculated from a 20-m-interval contour map, digitized from a 1970 aerial photography survey map with a 1:50,000 scale. A resolution of 30 m were defined for this DEM, as this resolution is recommended for the DEM-derived from a contour map with a 1:50,000 scale [22]. All the DEM derived variables were prepared as raster data with a resolution of 30 m. All these variables were prepared using UTM coordinates. The Geographic Information System (GIS) software ArcGIS, and ArcGIS extensions named Wetness Index 2.0 and Stream Orders were used for the derivation of the variables.

### Spatial lag model

To deal with the spatial dependence in the data, spatial lag models were used. The spatial lag model was modified from the ordinary regression model. The spatial lag model has a spatial component, which takes into account the spatial dependence in the dependent variable [23,24], as shown in Equation 1:

(1) *y *= *ρωy *+ *Xβ *+ *ε*

In this formula, y is the vector of dependent variable, *ρ *is the spatial autoregressive parameter determining the importance of spatial lag, *ω *is the structure of the assumed spatial dependency of the dependent variable, *β *is the vector of parameters, X is a matrix with observations on independent variables, and *ε *is the vector of errors.

In this equation, *ρωy *is a spatial lag term, which is essentially a weighted average of the neighboring values of the dependent variable. If the spatial autoregressive parameter (*ρ*) is significant, the spatial dependency does exist for the dependent variable. In this case, the spatial lag model can yield a more accurate description of the relationship between the dependent variable and the independent variables.

In spatial lag model, the spatial dependence between samples is typically expressed in a spatial weight matrix Wi, j, which consists of binary or generalized spatial weights assigned to the pairs of units i and j [25]. Two typical spatial weight matrices were used in this study: (1) a distance matrix (the weights are the inverse distance between samples), and (2) a binary contiguity matrix (the sample that is located within a threshold distance of another sample is assigned a value of 1, while the sample that is located beyond the threshold distance of another house is assigned a value of 0).

For the binary continuity matrix, a critical distance that defines the relevance of the nearby samples needs to be selected. Spatial weights can be defined based upon the distance of potential interaction or contiguity [14]. To identify this distance, the relevance of the nearby samples was examined by using Moran's I as a function of spatial distance. Moran's I indicates the degree of similarity between the values of the variable, and its value ranges approximately from +1 to -1 [26]. A positive Moran's I indicates spatial similarity among the samples, while a negative Moran's I indicates dissimilarity among the samples.

In this study, Moran's I were calculated using spatial distances ranging from 10 to 500 m with an increment of 20 m. A significance envelope of Moran's I were calculated using the Monte Carlo test. The increasing differences between the Moran's I and simulated envelope indicate an increasing level of spatial dependency in the samples. The distance, at which the samples have highest level of spatial dependence (i.e., Moran's I reaches the maximum value) were selected as the critical distance for the binary matrix.

### Data analysis

To investigate the relationship between adult mosquitoes and mosquito breeding habitats, three types of regression analyses were performed: ordinary regression, spatial lag model with the distance matrix, and spatial lag model with the binary matrix. The mosquito abundance data were log-transformed (ln (x+1)) to reach a normal distribution assumed by regression analysis. For each of the six types of habitat locations, its relationship with adult mosquitoes was investigated using each of the three types of regression analysis. The mosquito abundance was used as the dependent variable, and distance variables were used as the independent variable. In total, 18 regression models were created to describe the relationship between adult mosquitoes and each type of mosquito breeding habitats.

To develop a model that predicts the distribution of adult mosquitoes, the aforementioned three regression analyses were applied. For each regression analysis, mosquito abundance was used as the dependent variable. Six variables that represent the availability of mosquito breeding habitats and five environmental variables (Table 1) were used as independent variables. The independent variables were tested for heteroskedasticity and multicollinearity to satisfy the basic assumptions of regression analysis. If two variables are correlated, the one that explains less variability in the dependent variable is removed. Three models were created to predict the distribution of adult mosquitoes. The model development was accomplished in GeoDa [27].

Adjusted R square, Akaike Information Criterion (AIC), and Moran's I of regression errors were used for the model evaluation Three regression parameters, adjusted R square, Akaike Information Criterion (AIC) and Moran's I of regression errors, were used to aid the selection of the model that is best fitted to the data from the 21 models. The adjusted R square quantifies the amount of variation of the independent variable that is explained by the model, and it is often used in estimating the fit of the model to the data. The model with the largest R square is usually considered as the best model. The only problem with R square is that this parameter is not sensitive to overfitting, a problem caused by the involvement of too many variables in the model. When there are more variables added to the model, R square usually gets larger, which indicates that the model explains more variance. AIC serves as a useful supplement to R square. It not only estimates how well a model fits and but also penalizes the loss of degrees of freedom. This penalty discourages overfitting. The AIC test is considered the most reliable criteria for model fitting. The model with the smallest AIC value is considered to be the model that is best fitted to the data. The Moran's I test of the regression errors is a standard test to determine whether the developed regression model satisfies the independence assumption of the model residuals. The spatially dependent residuals indicate that the model does not satisfy a fundamental assumption of regression and it may be inefficient or wrong.

## Results

### Six types of habitat locations

A total of 6,612 water bodies was observed during the six field surveys. Among the all these water bodies, 32.15% of sites (2,126 sites) were identified as *An. gambiae *breeding sites. The numbers of pixels associated with each of six types of locations are provided in Table 2. On the integrated habitat map, the values of 1,321 pixels are larger than zero. In other words, breeding habitats were discovered at least once during six field surveys in the locations represented by these 1,321 pixels. Also on the integrated habitat map, the values of 632 pixels are equal to one, the values of 698 pixels are larger than one, and the values of 75 pixels are larger than two. Out of the 1,321 pixels, 372 of them contain breeding habitats that were observed in May 2003. Out of the 698 pixels, 143 pixels contain habitats that were observed in May 2003. The average distances between these six types of locations and the high-order streams are shown in Table 2. The locations where habitats were repeatedly observed have the shortest average distance to the high-order streams among the six types of locations. The standard deviation in their distances to the high-order streams is also the smallest among the six types of locations.

**Table 2:** Table showing the numbers of pixels that were occupy by six types of habitats locations, and the average and standard deviation for the distances between these six types of locations and the high-order streams.

**Habitat locations**	**Pixel number**	**Average distance **(m)	**Standard deviation**
locations where habitats were observed in May 2003	372	578	569
locations where habitats were observed at least once during six surveys	1321	621	513
locations where habitats were observed only once during six surveys	632	735	688
locations with repeatedly observed habitats	698	520	446
locations where habitats were observed at least three times during six surveys	75	877	846
locations where habitats were observed in May 2003 and at least once during other time periods	143	531	543

### Spatial dependence in adult mosquito abundance

**Figure 2:**
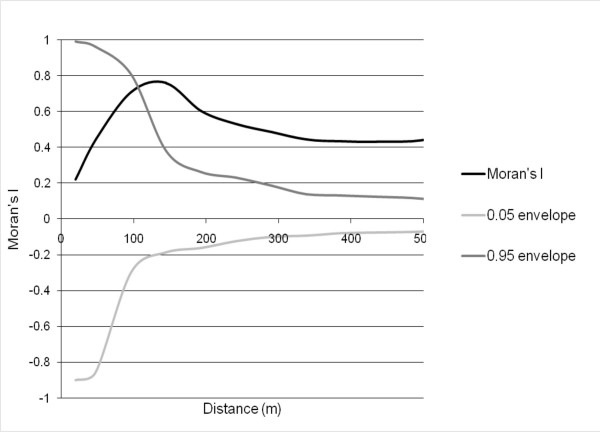
**Moran's I of the mosquito abundance data.** The black line is the calculated Moran's I. The grey lines delimit the 95% confidence envelope from the Monte Carlo simulation. Note that if the black line is above the grey lines, it indicates a spatial dependence in adult mosquito abundance.

*An. gambiae s.l. *and *An. funestus *are the two primary mosquito species in the area. The collected mosquitoes were mainly *An. gambiae s.l.*, only 6% of them were *An. funestus*. The data on *An. funestus *were not included. In May 2003, the numbers of *An. gambiae s.l. *captured in each house ranged from 0 to 161, with an average of 27. A correlogram for the Moran's I on the abundance of adult mosquitoes is presented in Figure 2. The correlogram were also compared with the Moran's I significance envelope, calculated using a Monte Carlo test. The dashed lines show the upper and lower bounds of the Moran's I envelope at 95% confidence intervals. The part of the black line that is above the dashed lines indicates the existence of spatial dependency of mosquito abundance (Figure 2).

As shown in Figure 2, the values of Moran's I for the abundance of adult mosquitoes are all positive. These results indicates that, at all distances, the numbers of mosquitoes in nearby houses are similar. The observed Moran's I increases with increasing distances, and the line of Moran's I intersects the simulated upper envelope at 100 m. This indicates that the distribution pattern of the adult mosquitoes is random at separation distances below 100 m. This line continues its increasing trend until the distance reaches 140 m, at which the highest Moran's I value (0.76) is obtained. After a distance of 140 m, the Moran's I value has a decreasing trend when the distance increases. This indicates that the spatial dependence in the abundance of adult mosquitoes is the most significant at the separation distance of 140 m.

### Relationship between adult mosquitoes and mosquito breeding habitats

Based on regression analysis, distance to locations with repeatedly observed habitats is the only distance variable that has a significant relationship with the abundance of adult mosquitoes. This variable is significant in any of the three regression analyses. The model parameters of these three regression analyses (i.e., adjusted R square, AIC, Moran's I of the residuals, regression coefficients, and probabilities) are shown in Table 3.

**Table 3:** Adjusted R2, AIC, Moran's I of regression residuals of the regression models that explain the relationship between adult mosquitoes and mosquito breeding habitats with repetitive occurrence.

**Spatial Lag Distance**			**Spatial Lag Binary**			**Ordinary Regression**		
**R2 = 0.45**			**R2 = 0.64**			**R2 = 0.17**		
**AIC = 271**			**AIC = 178**			**AIC = 333**		
**Morans' I = 0.21**			**Morans' I = 0.04**			**Morans' I = 0.62**		

Variable	Coefficient	P	Variable	Coefficient	P	Variable	Coefficient	P

Dist to habi	-9.50E-04	0.00	Dist to habi	-8.00E-04	0.00	Dist to habi	-1.90E-04	0.00
Constant	0.82	0.00	Constant	0.63	0.00	Constant	8.37	0.00
Lag	0.66	0.00	Lag	0.69	0.00	X	X	X

The coefficient and probability of significant variables are also listed.

X stands for None. Lag stands for the spatial lag term. Dist to habi stands for distance to mosquito breeding habitats with repetitive occurrence. The variables that have a significance level higher than 0.1 is considered insignificant.

Based on these three regression analyses, distance to locations with repeatedly observed habitats is negatively related to the abundance of adult mosquitoes. This indicates that the houses close to locations where habitats were repeatedly observed have more abundant adult mosquitoes. Among the three models, the ordinary regression model has the smallest adjusted R square, the largest AIC, and the largest Moran's I for the residuals. The spatial lag model with the binary matrix has the largest adjusted R square, the smallest AIC, and the smallest Moran's I for the residuals. The Moran's I for the residuals of the ordinary regression models is above 0.5, indicating the presence of significant spatial dependence in the residuals. The Moran's I for the spatial lag models with the binary and distance matrix are all smaller than 0.2. This implies that the residuals of the spatial lag models are independent of each other, which satisfies the fundamental assumption on the independence of the model errors.

### Models predicting distribution of adult mosquitoes

Three regression analyses on the relationship between adult mosquitoes and predictive variables indicate that distance to high-order streams is negatively related to the abundance of adult mosquitoes. This indicates that the houses with a great proximity to high-order streams have more abundant mosquitoes. Two of the three regression analysis agree that wetness index is negatively related to the abundance of adult mosquitoes. This indicates that the houses with higher levels of soil moisture and higher elevations have fewer mosquitoes. The lag components are significant in the lag model with the binary matrix and the lag model with the distance matrix. Adjusted R square, AIC, Moran's I of the residuals, regression coefficients, and probabilities of regression analyses are shown in Table 4.

**Table 4:** Adjusted R square, AIC, Moran's I of regression residuals of the regression models that predict the distribution of adult mosquitoes.

**Spatial Lag Distance**			**Spatial Lag Binary**			**Ordinary Regression**		
**R2 = 0.66**			**R2 = 0.74**			**R2 = 0.62**		
**AIC = 167**			**AIC = 111**			**AIC = 180**		
**Morans' I = 0.17**			**Morans' I = 0.06**			**Morans' I = 0.66**		

Variable	Coefficient	P	Variable	Coefficient	P	Variable	Coefficient	P

Wetness	-1.70E-02	0.09	X	X	X	Wetness	-2.00E-02	0.01
Dist to High	-3.40E-04	0.00	Dist to High	-2.60E-04	0.00	Dist to High	-3.70E-04	0.00
X	X	X	X	X	X	Elevation	-4.70E-03	0.03
Constant	29.03	0.00	Constant	0.83	0.00	Constant	8.37	0.00
Lag	0.61	0.00	Lag	0.45	0.00	X	X	X

The coefficient and probability of significant variables are also listed.

X stands for None. Lag stands for the spatial lag term. The variables that have a significance level higher than 0.1 is considered insignificant.

As shown in Table 4, among the three models explaining the distribution of adult mosquitoes, the ordinary regression model has the smallest adjusted R square, the largest AIC, and the largest Moran's I for the residuals. The spatial lag model with the binary matrix has the largest adjusted R square, the smallest AIC, and the largest Moran's I for the residuals.

The Moran's I for the residuals of the ordinary regression model is above 0.5, indicating the presence of significant spatial dependence in the residuals. The Moran's I for the four spatial lag models with either the binary or distance matrix are all smaller than 0.2 (Table 4). This implies that the residuals of the spatial lag models are independent of each other, which satisfies the fundamental assumption on the independence of the model errors.

The spatial lag model with the binary matrix has a adjusted R square that is larger than that of the ones with the distance matrix. This indicates that the model with the binary matrix contributes more to the explanation of the variance in the adult mosquito abundance than the spatial lag model with the distance matrix. This implies that the spatial dependence in the adult mosquito samples may be better demonstrated by the binary matrix.

## Discussion

### Spatial dependence in female adult mosquito observations

In this study, the models with the binary matrix always outperformed the models with the distance matrix. This indicates that the binary matrix may be more effective in depicting the spatial dependence in female adult mosquito abundance. If the binary matrix accurately depicts the spatial dependence in adult mosquito abundance, the mosquito observation at one location is influenced by all other observations within 140 m in a similar way. If all of the 2500 houses are evenly distributed, the distance between houses is 80 m. Therefore, each house is influenced by at least four houses around it. This implies that if the number of female mosquitoes is high in one house, four or more nearby houses may also have high numbers of female mosquitoes.

### Relationship between adult mosquitoes and mosquito breeding habitats

This study suggests that locations where habitats were repeatedly observed have a significant relationship with the distribution of adult mosquitoes. This indicates that habitats in these locations have a stronger relationship with adult mosquitoes than other habitats. As mentioned previously, the locations with repeatedly observed habitats have a greater proximity to high-order streams compared with other habitat locations (Table 2). One possible explanation is that high-order streams often locate in valley floors, which collect all run-off water in various depressions. The accumulation of run-off waters help maintains habitats in valley floors. The habitats in valley floors may be more stable than habitats in other areas.

This study suggests that the locations where habitats were only observed once during six surveys had no clear relationship with the adult mosquitoes. Since the adult mosquitoes were only surveyed once, it is difficult to exclude the possibility that these habitats may contribute to the adult mosquito population before or after the mosquito data were collected. Another possibility is that these habitats may not be productive. In other words, larvae failed to develop into pupa in these sites. Thus, these habitats had limited contribution to the adult mosquito population. There are a variety of reasons that a site may be unproductive. For example, it has been observed that some mosquito habitats may dry up easily in a few days if there is no rain [7]. Larvae at some habitats may also be washed away during heavy rainfalls [28]. Lack of nutrients at a habitat may also prevent the development of larvae [29]. Furthermore, this study suggests that the mosquito breeding habitats observed one week prior to the adult mosquito sampling time also have no significant relationship with the observed adult mosquitoes. Since the survey on adult mosquitoes was taken place only one week after the larval habitat survey, it is possible that some larvae in these sites did not have enough time to develop into the adults, and did not contribute to the adult mosquito populations when the adults were surveyed. It is also possible that some freshly emerged adult mosquitoes were still in the mating process.

The analysis indicates that the habitats that were observed at least three times have no significant correlation with adult mosquito distribution. The number of these habitats that were observed at least three times are probably too few to allow an reliable estimation of the statistical relationship between habitats and adult mosquitoes (these habitats consist of approximately 10% of all observed habitats).

### Relationship between adult mosquitoes and environmental variables

The results indicate that the houses with a great proximity to the high-order streams, which have five to six stream reaches, have more abundant mosquitoes. As mentioned previously, the high-order streams often locate in valley floors, where the habitats are provided with abundant rainy waters. It is worth noting that the distance to high-order streams is a better predictor of the distribution of adult mosquitoes compared with the distance to habitats with repetitive occurrences. This is probably due to the fact that the larvae were only surveyed six times in three years. Some habitats were absent during these surveys. The distance to high-order streams is more accurate in representing the habitat availability, as it describes the environmental conditions for habitats.

It is worth noting that the statistical relationship between low-order streams and the distribution of adult mosquitoes are found to be insignificant based on the analysis. This indicates that the streams that have three or less reaches may not have a strong influence on the distribution of adult mosquitoes. One possible explanation is that the low-order streams are usually temporal and may disappear after a short period of time [20]. Consequently, the habitats close to low-order streams might be influenced by the disappearance of the streams and exist only for a short period of time.

The comparison of the spatial and non-spatial models reveals notable differences in the relationship between adult mosquitoes and environmental variables. For example, the wetness index and the elevation are significant in the ordinary regression model, but insignificant in the spatial lag model with the binary matrix. These differences may reflect the bias in the results of the ordinary regression, since it is known that spatial dependence in the data can cause biases for the regression results. It has been pointed out that the possible biases include: (1) the failure to include important independent variables that are related to the dependent variable and (2) the retention of independent variables in the model as significant when they are not [27]. It seems that in this study the failure to account for spatial dependence in the dependent variable may have leaded to the adoption of the irrelevant variables, such as wetness index and elevation. In addition, the coefficient of the wetness in these two models is negative, indicating that the number of mosquitoes increases with the decreasing soil moisture level. The negative coefficient for the wetness index is counterintuitive, as it is generally believed that the mosquitoes prefer high levels of moisture [30].

**Figure 3:**
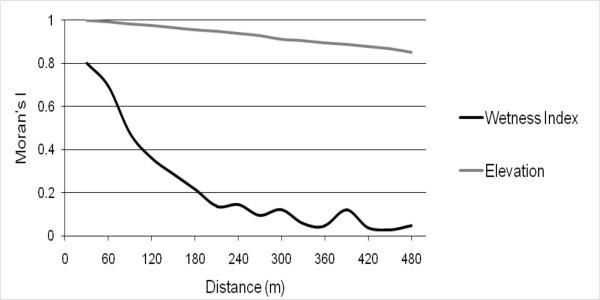
**Moran's I of Wetness Index and Elevation.** The Moran's I is calculated using the distances ranging from 30 to 480 m.

It has been suggested that the spatial dependence in the dependent variable is caused by the omitted variables that are spatially dependent [31]. However, few methods have been proposed to test whether the omitted variables truly cause the spatial dependence in the dependent variables. The difficulty lies in the fact that the omitted variables are omitted because of their inaccessibility. This study asserts that this variable may shed light on revealing the possible source of spatial dependence in the dependent variable. If a variable is significant in the non-spatial model, but insignificant in the spatial model, this variable may be related to a variable that causes the spatial dependence in the independent variable. For example, in this study, the soil moisture may be linked to one of reasons that caused the similarity in the adult mosquito observations. Figure 3 displays the Moran's I correlogram for wetness index (black line) and elevation (grey line), calculated using distances ranging from 30 to 480 m with a 30 m increment. The figure shows that the spatial dependence of the wetness index is clearly reduced after 100 m. For wetness index, the Moran's I at 150 m is 0.3. This may explain why the level of spatial dependence in adult mosquito abundance is the highest at 140 m (Figure 3). This figure also shows that the Moran's I of elevation is above 0.8 even when the distance reaches 480 m. Although this study suggests that the wetness index and elevation do not have a significant statistical relationship with adult mosquito abundance, the houses with a similar level of soil moisture and elevation may have conditions that are related to the number of adult mosquitoes. These conditions lead to spatial dependence in the adult mosquitoes. Because of these conditions, the numbers of mosquitoes in houses with similar soil moisture and elevation may be similar.

The adult mosquito and larval habitat data used in study were spatially extensive, and they were collected under the most typical climatic scenarios (rainy and dry seasons). However, the data used in this study also have several limitations. First, the larval habitats were only surveyed six times in three years, and density data were not used in the analysis. The availability, persistence and dimensions of mosquito larval habitats depend to a large extent on the frequency, duration and intensity of precipitation [28]. The larval density may also influence the distribution of the adult mosquitoes. However, the continues and consistent sampling of larval habitats is often resource prohibitive. Reliable data on larval density are also difficult to obtain, since the surface area of habitats varies considerably, and larvae are not evenly distributed in each habitat. Thus, only presence and absence data were used to represent the distribution of larval habitats.

Second, the adult mosquitoes were only surveyed once. The temporally extensive sampling of adult mosquitoes is preferred for mosquito studies, since the population of *An. gambiae s.l. *correlates closely with the seasonal rainfall patterns, and builds up rapidly and peak shortly after the onset of the rainy season [32]. This study only collected data on the adult mosquitoes in a rainy season, since the malaria outbreaks in the highland areas often follow rainy seasons [33]. Although this study indicates that it is possible to accurately estimate the distribution of female adult mosquitoes using environmental variables, further analysis is needed to determine whether the model developed in this study can be used to predict the distribution of adult mosquitoes outside rainy seasons.

Third, only environmental variables were used in this study. It is discovered that the distribution of adult mosquitos is likely to be affected by other variables, such as house roof type and bed net use [34]. Based on the field observations, the roofs of 80% of houses are made of iron-sheet roofing materials, and 10% of households use mosquito bed nets. The houses with iron-sheet roof or with bed nets are randomly distributed in the study. These two factors are less likely to have an influence on the distribution of adult mosquitoes. Thus, house roof type and bed net use were not considered in the analysis. However, caution must be practiced when the results of this study are applied in other areas where the house roof type and bed nets use is spatially heterogeneous.

In summary, this study identifies and quantifies the relationship between adult mosquitoes and mosquito breeding sites. It is found that the locations where habitats were repeated observed are significantly related to the distribution of female adult mosquitoes. The models predicting the distribution of female adult mosquitoes and their abundance were constructed. This study suggests that the houses in a great proximity to the streams with five or six reaches have more abundant adult mosquitoes than any other houses. Some studies also reported that mosquito breeding habitats tend to appear at the valley bottoms, where streams are usually located [35,36]. This study is the first attempt to introduce the stream orders in the mosquito studies. Distance to high-order streams can be easily generated from a DEM. The detailed field survey is not necessary for the acquisition of this variable. The study reveals that the ignorance of spatial dependence in the modeling can cause misrepresentation of the relationship between adult mosquitoes and explanatory variables. The findings of study are important for public health decision-making related to adult mosquito surveillance and malaria control.

## Competing interests

The authors declare that they have no competing interests.

## Authors' contributions

LL conceived the study, analyzed the data and drafted the manuscript. LB contributed to the conception of the study and provided expertise on environmental modeling. GY is the principle investigator of the project that collected the field data. All authors approved the final manuscript.
